# Circulating α-Klotho Is Positively Associated with Eosinophil Counts in Community-Dwelling Older Adults: A Cross-Sectional Study

**DOI:** 10.3390/biology15211916

**Published:** 2026-10-22

**Authors:** Aigul Abduldayeva, Gulnur Doszhanova, Nikolay Safonov, Aigul Khaldenova, Olzhas Kozhamkulov, Aida Shuakbayeva, Gulbanu Boranbayeva, Ainagul Kazbekova, Altyn Moldabayeva, Elvira Nagyzbekkyzy, Gulziya Bidatova, Zhanar Kalmakova

**Affiliations:** 1 Scientific Research Institute of Preventive Medicine Named After Academician Y.D. Dalenov, Astana Medical University, Astana 010000, Kazakhstanaidapvl76@gmail.com (A.S.);; 2 Department of Internal Medicine No. 1, Astana Medical University, Astana 010000, Kazakhstan; kazbekova.a@amu.kz; 3 Department of Internal Medicine No. 2, Astana Medical University, Astana 010000, Kazakhstan; moldabaeva.a@amu.kz (A.M.); nagyzbekkyzy.e@amu.kz (E.N.); 4 National Center of Public Healthcare of the Ministry of Health of the Republic of Kazakhstan, Astana 010000, Kazakhstan; zha_kalmakova@mail.ru

**Keywords:** α-Klotho, eosinophils, leukocyte differential, older adults, ELISA, assay calibration, exploratory analysis, Kazakhstan

## Abstract

**Background:** The relation of circulating α-Klotho to individual leukocyte populations is uncertain. An exploratory 13-marker hematological screen identified eosinophils as a candidate signal, motivating this focused analysis. **Methods:** The grant-funded α-Klotho substudy included 504 assayed serum specimens. After excluding one participant aged < 60 years and one with missing age, 502 participants were eligible. α-Klotho was measured with the FineTest Human SAKL ELISA kit (EH4278; calibration range 93.75–6000 pg/mL). Because results above the upper calibrator had been software-extrapolated without repeat dilution, the primary analysis was restricted to 397 participants with reported concentrations ≤ 6000 pg/mL; 388 had an eosinophil count. Spearman’s correlations were corrected across 13 hematological markers using Benjamini–Hochberg adjustment. Ordinary least-squares models used HC3 heteroskedasticity-consistent standard errors. **Results:** Median α-Klotho in the primary cohort was 1179.67 pg/mL (IQR 920.07–1686.51). Absolute eosinophil count correlated weakly with α-Klotho (ρ = 0.120; *p* = 0.018), but the association did not survive correction across the screening family (q = 0.146). In the fully adjusted model (*n* = 376), each 1 SD higher log-eosinophil count was associated with 8.4% higher α-Klotho (95% CI 2.0–15.2%; *p* = 0.009). The quartile trend was significant (*p* = 0.019), whereas median regression produced a smaller estimate (5.4%; bootstrap 95% CI −0.6 to 12.5%). Estimates were similar after recruitment-stratum adjustment and after excluding eosinophilia, chronic respiratory disease, or leukocyte counts > 15 × 10^9^/L. **Conclusions:** Within the assay calibration range, higher eosinophil counts were associated with modestly higher serum α-Klotho after adjustment for measured covariates. The post hoc origin, assay limitations, and cross-sectional design make this a hypothesis-generating immune association rather than evidence of causality, healthy longevity, or a clinical threshold.

## Simple Summary

Alpha-Klotho is a protein involved in processes related to aging, kidney function, and inflammation. Eosinophils are white blood cells commonly linked to allergy and parasitic infection, but they may also support tissue repair. We examined whether blood alpha-Klotho levels were related to eosinophil counts in older adults attending active aging centers in Astana, Kazakhstan. The main analysis included 397 participants whose alpha-Klotho measurements fell within the test kit’s stated measurement range. Higher eosinophil counts were associated with modestly higher alpha-Klotho levels after accounting for age, sex, body size, kidney function, total white blood cell count, smoking, and chronic respiratory disease. However, the simple correlation did not remain significant after adjustment for the number of blood markers screened, and an analysis focused on the middle of the alpha-Klotho distribution was less certain. Because all variables were measured at one time and detailed information on allergy, parasitic infection, medication use, season, and time of blood collection was unavailable, the study cannot determine cause and effect. The finding should therefore be viewed as an initial observation requiring confirmation in independent studies with stronger laboratory quality control.

## 1. Introduction

The klotho gene was identified in a mouse model in which deficient expression produced shortened lifespan and age-related phenotypes [1]. Subsequent experimental work showed that Klotho overexpression extended lifespan in mice and that the extracellular domain of α-Klotho could circulate as a humoral factor [2]. In humans, soluble α-Klotho is derived predominantly from the kidney and participates in mineral metabolism, oxidative-stress responses, and multiple signaling pathways [3,4,5]. Observational studies have linked lower circulating α-Klotho to adverse outcomes in some older populations, although concentration estimates and clinical associations vary across assays and cohorts [6,7,8,9].

Most population studies of α-Klotho and inflammation have emphasized composite inflammatory indices rather than individual leukocyte lineages [10,11,12,13]. Eosinophils are of interest because their biology extends beyond allergic inflammation and parasitic infection: tissue eosinophils can participate in epithelial maintenance, immune regulation, and repair [14,15]. A peripheral-blood eosinophil count, however, cannot distinguish inflammatory from tissue-homeostatic phenotypes.

In a US NHANES analysis, Han and colleagues reported lower eosinophil counts at higher α-Klotho concentrations [16]. Whether the same pattern is observed in older Central Asian adults is uncertain. Population context, renal function, respiratory phenotype, unmeasured exposures, and important differences between commercial α-Klotho immunoassays may all affect observed associations [8,9].

The eosinophil focus of the present study arose after an exploratory screen of 13 hematological markers in a grant-funded α-Klotho substudy. The primary objective was therefore to characterize, rather than confirm, the association between serum α-Klotho and absolute eosinophil count. Eosinophil percentage and the eosinophil-to-lymphocyte ratio (ELR) were supportive measures. Because the data were cross-sectional, the regression direction was selected for interpretability and was complemented by a reverse-direction sensitivity model; neither specification establishes temporal direction.

## 2. Materials and Methods

### 2.1. Study Design and Participants

This secondary cross-sectional analysis used de-identified data from community-dwelling adults attending the municipal Active Aging Centre network in Astana, Kazakhstan. The α-Klotho component was a laboratory substudy within a larger multidisciplinary state-funded grant project. The substudy included all 504 serum specimens assayed under the prospectively defined budgeted allocation. Participants enrolled for other project objectives but not allocated to α-Klotho testing were outside the sampling frame for this substudy and were not classified as having missing α-Klotho measurements. One participant aged 58 years and one participant with missing age were excluded, leaving 502 eligible participants aged ≥ 60 years.

**Figure 1:**
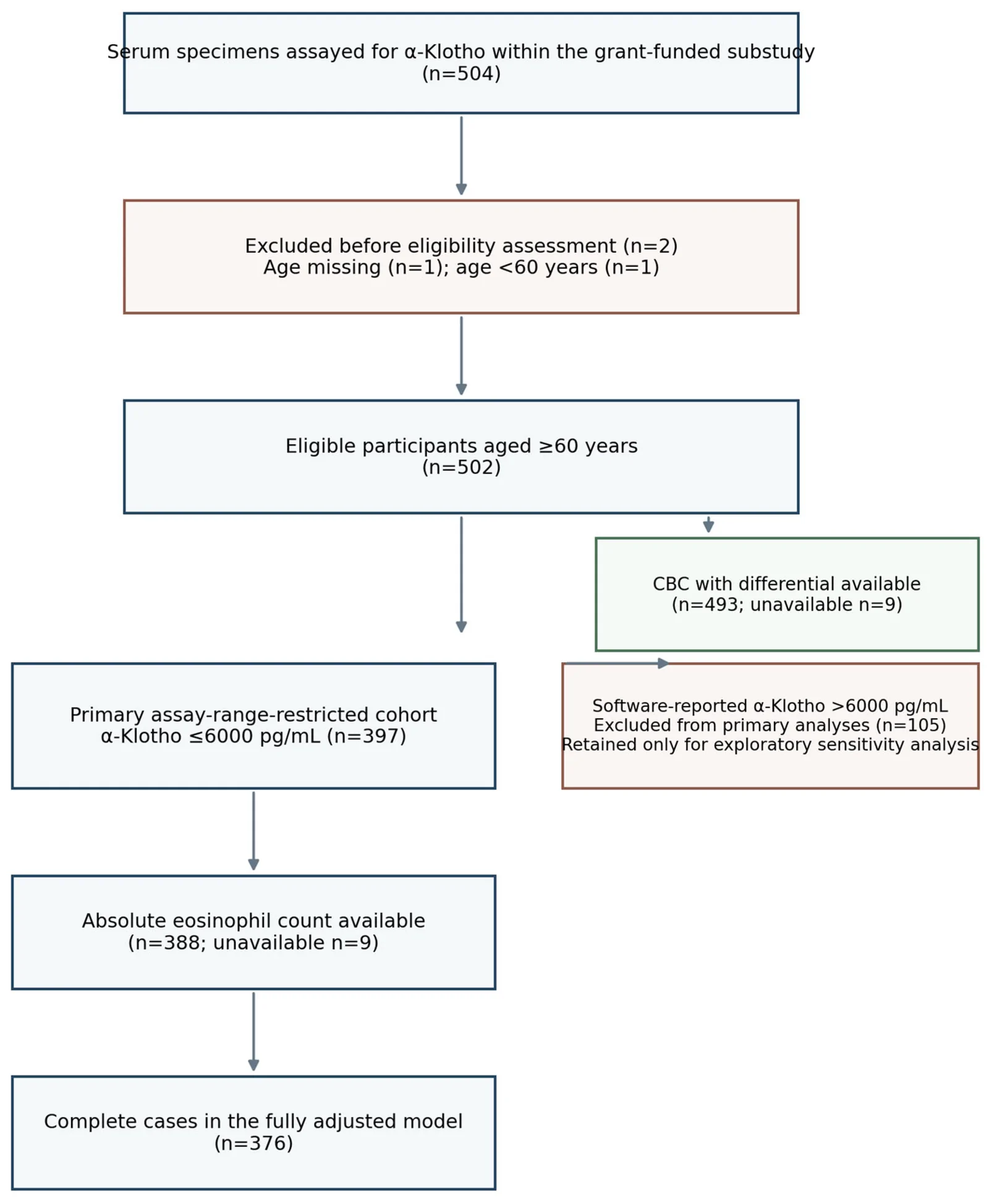
Flow diagram of the grant-funded α-Klotho substudy and analytical samples. Note. CBC with differential was available for 493 of 502 eligible participants. Model 1 and Model 2 used 388 participants with an absolute eosinophil count; Model 3 used 376 complete cases. Arrows indicate participant flow. Blue boxes identify the source and analytical cohorts, brown boxes identify exclusions, and the green box indicates CBC availability.

The FineTest assay calibration range ended at 6000 pg/mL. Because concentrations above that value had been generated by software extrapolation without additional dilution and remeasurement, the primary analytical cohort was restricted to reported α-Klotho concentrations ≤ 6000 pg/mL (*n* = 397). The 105 eligible participants with software-reported values > 6000 pg/mL were retained only for an explicitly exploratory sensitivity analysis. Absolute eosinophil counts were available for 388 participants in the primary cohort, and 376 had complete data for the fully adjusted model (Figure 1). Missing values were not imputed.

The parent study enrolled older adults able to provide written informed consent and complete study assessments. Acute illness, severe cognitive impairment preventing questionnaire completion, and incomplete core assessments were exclusion criteria in the parent protocol.

### 2.2. Serum α-Klotho Measurement

Soluble α-Klotho was measured in serum by solid-phase double-antibody sandwich enzyme-linked immunosorbent assay using the FineTest Human SAKL (Soluble alpha-Klotho) ELISA Kit (Wuhan Fine Biotech Co., Ltd., Wuhan, China; catalog EH4278; lot FN260225; 96 tests) [17]. Serum had been stored at −20 °C for up to six months and underwent one freeze–thaw cycle. Samples were diluted 1:2 by mixing 60 µL serum with 60 µL kit sample-dilution buffer. Optical density was read on a Digital Analytics Systems A4 microplate reader at 450 nm with 620 nm as the reference wavelength, and concentrations were calculated from the plate standard curve.

The manufacturer-stated calibration range was 93.75–6000 pg/mL and the stated sensitivity was 56.25 pg/mL; separate study-specific lower and upper limits of quantification were not supplied by the laboratory. The manufacturer’s validation documentation reports dilution-linearity recoveries of 91–103% at 1:2, 83–101% at 1:4, and 83–99% at 1:8, with intra-assay coefficients of variation of 4.70–5.36% and inter-assay coefficients of variation of 5.15–5.26% [17]. These are manufacturer data and not quality-control estimates from the present study. Six 96-well plates were run in six analytical sessions from 12 to 25 June 2026. Values above 6000 pg/mL were extrapolated by the instrument software; no additional dilution or repeat measurement was performed. Four specimens were measured in duplicate during initial assay evaluation; in the available raw run file, duplicate optical-density coefficients of variation ranged from 1.1% to 7.2%. Formal study-wide intra-assay and inter-assay coefficients of variation were not available. These limitations motivated the assay-range restriction used for the primary analysis.

### 2.3. Hematological Measurements

Complete blood counts with five-part leukocyte differentiation were performed using a Mindray BC-5800 automated hematology analyzer (Shenzhen Mindray Bio-Medical Electronics Co., Ltd., Shenzhen, China). The leukocyte differential was determined by flow cytometry. Variables included total leukocytes, platelets, and absolute neutrophil, lymphocyte, monocyte, eosinophil, and basophil counts, together with eosinophil percentage. Erythrocyte sedimentation rate (ESR) was measured using the Panchenkov method. ELR was calculated as absolute eosinophils divided by absolute lymphocytes; infinite values were treated as missing.

### 2.4. Covariates

Age, sex, body mass index (BMI), smoking status, coded recruitment stratum, and self-reported chronic respiratory disease (bronchial asthma or chronic obstructive pulmonary disease) were obtained from the database. The recruitment-stratum codes combined district branch and attendance session or time window within the same municipal Active Aging Centre network; they did not represent independent research centers. Estimated glomerular filtration rate (eGFR) was calculated from serum creatinine, age, and sex with the 2021 CKD-EPI creatinine equation without race [18]. Total leukocyte count was included to distinguish an eosinophil-specific association from overall leukocytosis. Available comorbidity fields combined cardiovascular disease (hypertension or ischemic heart disease), endocrine disease (diabetes, obesity, or thyroid disease), and urinary-system disease (chronic kidney disease, glomerulonephritis, pyelonephritis, or cystitis); these grouped variables were used descriptively and in an extended sensitivity model.

### 2.5. Statistical Analysis

Continuous variables were examined for distributional shape. Approximately symmetric variables are summarized as mean ± standard deviation (SD), right-skewed variables as median and interquartile range (IQR), and categorical variables as *n* (%). The primary univariable analysis used Spearman’s rank correlation between α-Klotho and absolute eosinophil count. Eosinophil percentage and ELR were supportive measures. The original screening family contained 13 hematological measures: total leukocytes; absolute neutrophils, lymphocytes, monocytes, eosinophils, and basophils; eosinophil percentage; ESR; neutrophil-to-lymphocyte ratio; platelet-to-lymphocyte ratio; systemic immune-inflammation index; systemic inflammation response index; and ELR. Benjamini–Hochberg correction was applied across this family [19]. Because the eosinophil focus was selected from the screen, all subsequent *p* values are interpreted as exploratory rather than confirmatory.

For regression, the outcome was log_10_(α-Klotho) and the exposure was ln(absolute eosinophils +0.01), standardized using the eligible 502-participant cohort (1 SD = 0.612). Ordinary least-squares regression used HC3 heteroskedasticity-consistent standard errors. Model 1 was unadjusted; Model 2 adjusted for age and sex; Model 3 additionally adjusted for BMI, eGFR, total leukocyte count, smoking status, and chronic respiratory disease. Coefficients were back-transformed to percentage differences in α-Klotho per 1 SD higher log-eosinophil count.

The shape and robustness of the association were examined using adjusted eosinophil quartiles with a trend test, median regression with 1000 bootstrap replicates, adjustment for coded recruitment stratum, exclusion of eosinophilia (>0.5 × 10^9^/L), exclusion of chronic respiratory disease, exclusion of total leukocyte counts > 15 × 10^9^/L, and additional adjustment for the available grouped comorbidities. A reverse-direction model used log-eosinophils as the outcome and standardized log_10_(α-Klotho) as the exposure. Effect modification was explored for sex, age (60–69 versus ≥70 years), respiratory disease, and eGFR (<60 versus ≥60 mL/min/1.73 m^2^) by adding product terms and reporting stratified estimates. Clustering of values > 6000 pg/mL across coded recruitment strata was assessed using the Pearson chi-square statistic with a permutation *p* value based on 10,000 label permutations because several strata were small. The full set of software-reported α-Klotho values was analyzed only as a sensitivity analysis because values above the calibration range had not been analytically verified. Analyses were performed in Python 3.12.14 using NumPy 2.3.5, pandas 2.2.3, SciPy 1.17.0, and scikit-learn 1.8.0. Two-sided *p* < 0.05 was used for nominal inference; q < 0.05 denoted significance after screening-family correction. Reporting followed STROBE principles [20].

### 2.6. Ethical Considerations

The study was conducted in accordance with the Declaration of Helsinki. Ethical approval was granted by the Local Ethics Committee of NJSC Astana Medical University (No. 3, 21 October 2024). All participants provided written informed consent before participation.

## 3. Results

### 3.1. Participant Flow and Assay-Range Restriction

Of 504 participants with an α-Klotho assay result in the grant-funded substudy, 502 met the age criterion. A complete blood count with leukocyte differential was available for 493 of these participants. The instrument software reported α-Klotho values above 6000 pg/mL for 105 of 502 eligible participants (20.9%); these values were excluded from the primary analysis. The resulting primary cohort contained 397 participants, including 388 with absolute eosinophil data and 376 complete cases for Model 3 (Figure 1). Values above 6000 pg/mL did not cluster across coded recruitment strata (χ^2^ = 6.19; permutation *p* = 0.736), but sample-to-plate identifiers were unavailable, so plate or analytical-run clustering could not be tested.

### 3.2. Participant Characteristics

In the primary assay-range-restricted cohort (*n* = 397), the mean age was 68.2 ± 5.0 years and 329 participants (82.9%) were women. Median serum α-Klotho was 1179.67 pg/mL (IQR 920.07–1686.51), median absolute eosinophil count was 0.13 × 10^9^/L (IQR 0.09–0.19), and median eosinophil percentage was 2.40% (IQR 1.70–3.20) (Table 1).

### 3.3. α-Klotho and Eosinophil Measures

Absolute eosinophil count had a small positive rank correlation with α-Klotho (ρ = 0.120; *p* = 0.018), but this association did not meet the false-discovery-rate threshold across the 13-marker screening family (q = 0.146). Eosinophil percentage and ELR were not significant in the assay-range-restricted cohort (Table 2). No measure in the screening family met q < 0.05 (Table S1). Serum α-Klotho was not significantly correlated with eGFR (ρ = −0.068; *p* = 0.179; *n* = 391).

**Figure 2:**
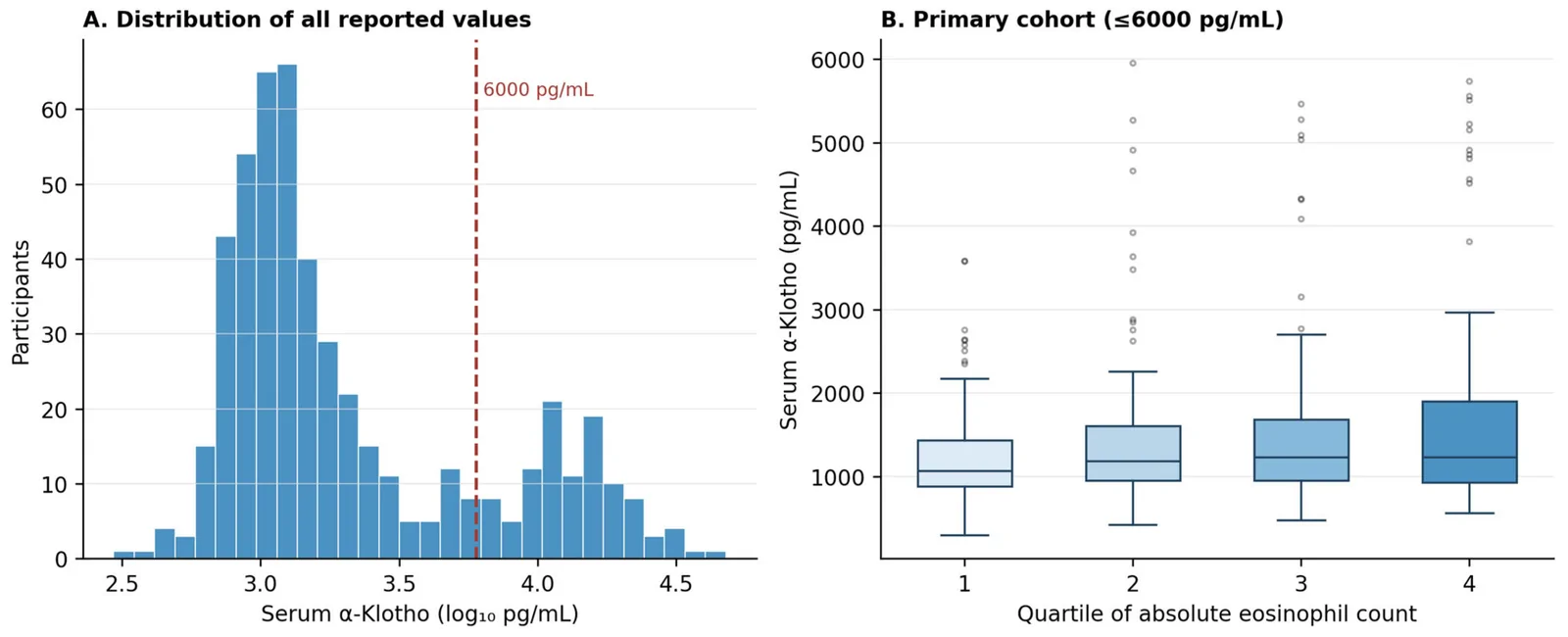
Distribution of serum α-Klotho and its pattern across eosinophil quartiles. Note: Panel (**A**) includes all 502 eligible software-reported values; the dashed line marks the upper assay calibrator (6000 pg/mL). Panel (**B**) includes only the primary assay-range-restricted cohort with an available eosinophil count (*n* = 388). Boxes show IQRs and center lines show medians. Circles indicate observations beyond the whiskers (more than 1.5 interquartile ranges below the first quartile or above the third quartile).

The distribution of all software-reported α-Klotho values and the quartile pattern within the calibration range are shown in Figure 2. In the primary cohort, median α-Klotho rose from 1063.16 pg/mL in the lowest eosinophil quartile to 1231.70 pg/mL in the highest quartile. In the fully adjusted categorical model, Q4 had 18.7% higher α-Klotho than Q1 (95% CI 2.0–38.1%; *p* = 0.027), with a significant linear trend across quartiles (*p* = 0.019; Table S2).

### 3.4. Multivariable Association

In the unadjusted model, each 1 SD higher log-eosinophil count was associated with 9.6% higher α-Klotho (95% CI 3.1–16.4%; *p* = 0.003). The estimate changed little after adjustment for age and sex. In Model 3 (*n* = 376), the corresponding adjusted difference was 8.4% (95% CI 2.0–15.2%; *p* = 0.009) after accounting for BMI, eGFR, total leukocyte count, smoking, and chronic respiratory disease (Table 3).

### 3.5. Sensitivity, Distributional, and Reverse-Direction Analyses

**Figure 3:**
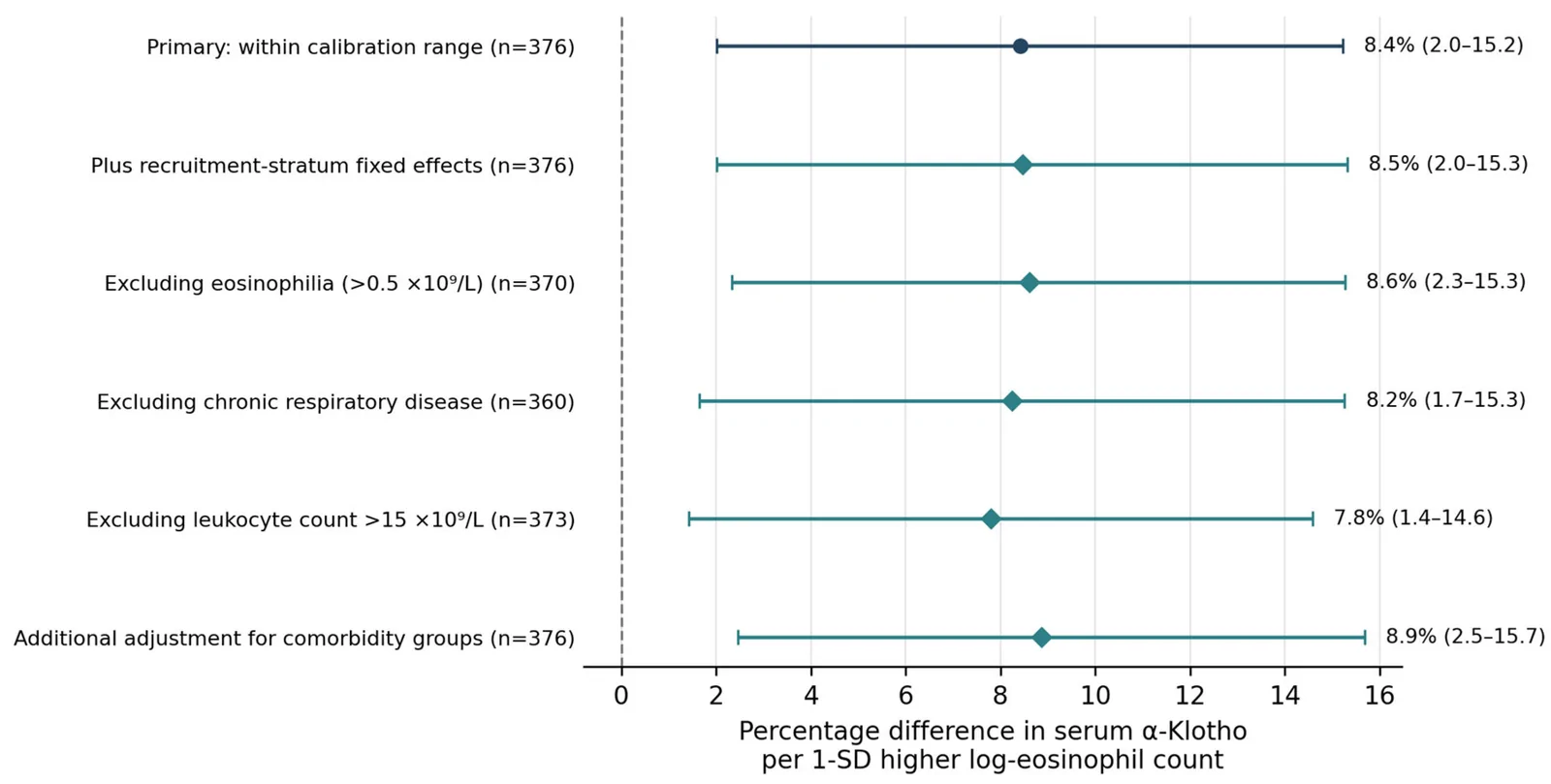
Primary and sensitivity estimates within the assay calibration range. Note: Points show the percentage difference in serum α-Klotho per 1 SD higher log-eosinophil count; bars show 95% HC3 confidence intervals. The exploratory all-values analysis is omitted from the figure.

The adjusted estimate remained between 7.8% and 8.9% after recruitment-stratum adjustment; exclusion of eosinophilia, chronic respiratory disease, or leukocyte counts > 15 × 10^9^/L; and additional adjustment for available comorbidity groups (Table 4 and Figure 3). Median regression yielded a smaller and less precise central-shift estimate of 5.4% (bootstrap 95% CI −0.6 to 12.5%). When all software-reported values were analyzed, including those above the calibration range, the estimate was 22.3% (95% CI 9.1–37.0%); because these high values were not verified by dilution and repeat measurement, this result was treated as exploratory and was not used for the principal conclusion. In the reverse-direction model, each 1 SD higher log_10_(α-Klotho) was associated with 19.1% higher eosinophil count (95% CI 4.4–35.9%; *p* = 0.009), supporting association but not temporal direction.

### 3.6. Effect Modification

There was no statistical evidence that the adjusted association differed by sex (*p* for interaction = 0.774), age group (*p* = 0.908), chronic respiratory disease (*p* = 0.632), or eGFR category (*p* = 0.868). Stratified estimates were imprecise in men, participants with respiratory disease, and participants with eGFR < 60 mL/min/1.73 m^2^ because these strata were small (Table S3).

## 4. Discussion

### 4.1. Principal Findings

In this exploratory study of community-dwelling older adults, absolute eosinophil count showed a small positive association with serum α-Klotho within the manufacturer-stated calibration range of the assay. The rank correlation was nominally significant but did not survive correction across the original 13-marker screening family. By contrast, the association remained statistically significant in adjusted mean-based models and across several sensitivity analyses. The fully adjusted estimate was modest—8.4% higher α-Klotho per 1 SD higher log-eosinophil count—and median regression was weaker and compatible with no central shift. These findings support a hypothesis for replication, not a confirmatory biomarker claim.

The quartile analysis suggested an ordered pattern, but the larger Q4-versus-Q1 difference and the weaker median-regression estimate indicate that the association may be more evident in the upper part of the within-range α-Klotho distribution than at its center. This distributional feature is important when interpreting a mean-based model. Neither the forward nor reverse regression establishes directionality.

### 4.2. Comparison with Previous Evidence and Assay Context

The positive direction differs from the NHANES analysis reported by Han et al. [16]. NHANES used an IBL International ELISA with extensive duplicate testing and quality control; laboratory documentation reported an observed range of 151.3–2605.1 pg/mL [21]. In contrast, this study used FineTest EH4278, for which the manufacturer states a calibration range of 93.75–6000 pg/mL [17]. A published study using EH4278 in older adults with diabetes and limb ischemia reported a mean α-Klotho concentration of 403.1 ± 179.3 pg/mL [22]. Our within-range median of 1179.67 pg/mL remains higher than that in those reports. Differences in population, specimen handling, dilution, calibration, quality-control procedures, and antibody specificity may contribute; absolute concentrations from different immunoassays and workflows should not be treated as directly interchangeable [8,9].

The absence of a significant α-Klotho-eGFR correlation in this cohort does not validate or invalidate the assay by itself, but it removes one expected internal biological association and reinforces the need for cautious interpretation. Because high values were not analytically verified, we did not attempt to explain them as a biological phenotype.

### 4.3. Biological Interpretation

Eosinophils are functionally heterogeneous. They participate in allergic and type 2 inflammation, yet tissue-resident populations also contribute to mucosal homeostasis and repair [14,15]. A routine peripheral count cannot determine which function predominates. Accordingly, the present association does not demonstrate a protective eosinophil phenotype, enhanced α-Klotho signaling, or a longevity mechanism. Unmeasured allergic disease, airway inflammation, medication use, infection, and environmental exposure could generate or modify the observed pattern.

### 4.4. Strengths and Limitations

Strengths include the analysis of an underrepresented Central Asian older population, focused leukocyte-differential question, explicit retention of the 13-marker multiplicity correction, adjustment for renal function and total leukocyte count, and concordant estimates after several clinically motivated restrictions. Restricting the principal analysis to the stated calibration range prevents unverified software extrapolations from determining the main conclusion.

The study also has substantial limitations. First, the cross-sectional design precludes temporal and causal inference, and the eosinophil focus was selected after the hematological screen. Second, 105 eligible results (20.9%) exceeded the upper calibrator. They were not diluted and remeasured, study-wide intra-assay and inter-assay CVs were unavailable, and sample-to-plate identifiers were not retained for batch modeling. Excluding these results reduced precision and may introduce selection; retaining them as exact concentrations would introduce a different, potentially larger measurement error. The primary restriction and exploratory all-values analysis should therefore be considered together.

Third, the database lacked detailed information on atopy, allergic rhinitis, asthma activity, parasitic infection, acute infection, systemic or inhaled corticosteroids, biologic therapy, and other medications affecting eosinophils. Opisthorchiasis and other helminth exposure are specific plausible confounders in parts of Kazakhstan, but individual testing or residence-based exposure data were unavailable. Blood-draw date, season, and time of day were also unavailable, preventing control for seasonal or circadian variation; a small healthy-volunteer pilot reported minimal daytime variation in soluble α-Klotho [23]. Analytical run dates are not a substitute for sampling dates. Fourth, the respiratory variable combined asthma and chronic obstructive pulmonary disease, and other comorbidity fields combined several diagnoses, limiting disease-specific adjustment. Fifth, the cohort was predominantly female and recruited from active aging centers, limiting generalizability. Finally, this study did not measure mortality, incident disease, frailty trajectory, or remaining life expectancy.

### 4.5. Implications for Future Research

Replication should use prospectively standardized blood-draw timing, sample-level plate identifiers, duplicate measurements, plate-level quality-control material, and serial dilution with repeat measurement for specimens above the calibration range. Detailed assessment of allergic disease, parasite exposure, corticosteroids and biologics, acute infection, kidney disease, and respiratory phenotype is required. Prospective follow-up would be necessary before relating a joint eosinophil-α-Klotho profile to mortality, frailty, or healthy-aging outcomes.

## 5. Conclusions

Within the stated assay calibration range, higher absolute eosinophil counts were associated with modestly higher serum α-Klotho after adjustment for measured covariates. The univariable signal did not survive correction across the original hematological screening family, and median regression was less precise. The result is therefore hypothesis-generating and requires independent replication with stronger analytical quality control. It does not establish causality, a protective eosinophil phenotype, a longevity mechanism, or a clinically meaningful α-Klotho or eosinophil threshold.

## Figures and Tables

**Table 1:** Characteristics of the primary assay-range-restricted cohort.

Characteristic	*n*	Value
Age, years	397	68.2 ± 5.0
Women	397	329 (82.9%)
Body mass index, kg/m^2^	396	28.5 ± 4.6
Serum α-Klotho, pg/mL	397	1179.67 [920.07–1686.51]
eGFR, mL/min/1.73 m^2^	391	84.0 ± 13.6
Leukocytes, ×10^9^/L	389	5.67 [4.80–6.58]
Neutrophils, ×10^9^/L	388	3.04 [2.42–3.63]
Lymphocytes, ×10^9^/L	388	2.03 [1.62–2.37]
Monocytes, ×10^9^/L	388	0.36 [0.28–0.44]
Eosinophils, ×10^9^/L	388	0.13 [0.09–0.19]
Eosinophils, %	388	2.40 [1.70–3.20]
Basophils, ×10^9^/L	388	0.02 [0.02–0.03]
Platelets, ×10^9^/L	389	216.00 [188.00–250.00]
Erythrocyte sedimentation rate, mm/h	386	10.00 [5.00–16.00]
Never smoked	387	348 (89.9%)
Former smoker	387	24 (6.2%)
Current smoker	387	15 (3.9%)
Cardiovascular disease (hypertension or ischemic heart disease)	387	218 (56.3%)
Endocrine disease (diabetes, obesity, or thyroid disease)	387	124 (32.0%)
Chronic respiratory disease (asthma or COPD)	387	16 (4.1%)
Urinary-system disease (CKD, glomerulonephritis, pyelonephritis, or cystitis)	387	11 (2.8%)
Coded recruitment stratum (branch/session group)	397	2: 30 (7.6%); 3: 20 (5.0%); 4: 11 (2.8%); 5: 38 (9.6%); 6: 23 (5.8%); 7: 4 (1.0%); 8: 8 (2.0%); 9: 242 (61.0%); 10: 3 (0.8%); 15: 18 (4.5%)

Note. Values are mean ± SD, median [IQR], or *n* (%), as appropriate. The α-Klotho primary cohort was restricted to reported concentrations ≤ 6000 pg/mL. Recruitment-stratum codes combine branch and attendance session/time window within one municipal Active Aging Centre network. COPD, chronic obstructive pulmonary disease; eGFR, estimated glomerular filtration rate.

**Table 2:** Correlations between serum α-Klotho and eosinophil-related measures.

Measure	*n*	Spearman ρ	*p*	BH q
Eosinophils, absolute	388	0.120	0.018	0.146
Eosinophils, %	388	0.077	0.131	0.339
ELR	388	0.084	0.100	0.325

Note. BH, Benjamini–Hochberg; ELR, eosinophil-to-lymphocyte ratio. q values were calculated across the complete 13-marker screening family shown in Table S1.

**Table 3:** Association of absolute eosinophil count with serum α-Klotho in the primary cohort.

Model	*n*	% Difference	95% CI	*p*
Model 1: unadjusted	388	9.6%	3.1 to 16.4	0.003
Model 2: age and sex	388	9.6%	3.1 to 16.6	0.004
Model 3: fully adjusted	376	8.4%	2.0 to 15.2	0.009

Note. The outcome was log_10_(α-Klotho). The exposure was ln(absolute eosinophils + 0.01), expressed per 1 SD. Model 2 adjusted for age and sex. Model 3 additionally adjusted for BMI, eGFR, total leukocyte count, smoking, and chronic respiratory disease. HC3 heteroskedasticity-consistent confidence intervals are shown.

**Table 4:** Sensitivity and distributional analyses.

Analysis	*n*	% Difference	95% CI	*p*
Primary: within calibration range	376	8.4%	2.0 to 15.2	0.009
Plus recruitment-stratum fixed effects	376	8.5%	2.0 to 15.3	0.010
Excluding eosinophilia (>0.5 ×10^9^/L)	370	8.6%	2.3 to 15.3	0.007
Excluding chronic respiratory disease	360	8.2%	1.7 to 15.3	0.014
Excluding leukocyte count >15 ×10^9^/L	373	7.8%	1.4 to 14.6	0.016
Additional adjustment for comorbidity groups	376	8.9%	2.5 to 15.7	0.006
All software-reported values (exploratory)	479	22.3%	9.1 to 37.0	<0.001
Median regression, fully adjusted	376	5.4%	−0.6 to 12.5	-

Note. All rows except median regression used OLS with HC3 covariance and the fully adjusted covariate set. The median-regression interval was obtained from 1000 bootstrap replicates. The all-values analysis includes software-extrapolated results >6000 pg/mL and is exploratory only.

## Data Availability

The de-identified data are not publicly available because they form part of an ongoing state-funded multidisciplinary grant project, and public deposition is not covered by the project’s current data-governance arrangements. Data supporting the findings of this article may be obtained from the corresponding authors on reasonable request, subject to approval by Astana Medical University and the project leadership, compliance with applicable data-protection requirements, and completion of an appropriate data-use agreement.

## Supplementary Materials

The following supporting information can be downloaded at https://www.mdpi.com/article/10.3390/biology15211916/s1: Table S1: Complete 13-marker hematological screening correlations with serum α-Klotho; Table S2: Adjusted eosinophil-quartile and reverse-direction analyses; Table S3: Stratified estimates and tests of effect modification.
