# Supplementary material for: Circulating α-Klotho Is Positively Associated with Eosinophil Counts in Community-Dwelling Older Adults: A Cross-Sectional Study
Source: Biology (Basel). 2026 Oct 22;15(21):1916. doi: 10.3390/biology15211916 (PMC13761448; doi:10.3390/biology15211916)
Supplement: Supplementary file 1 [file biology-15-01916-s001.zip › biology-4613093-supplementary.pdf]

Supplementary Materials

Circulating α-Klotho Is Positively Associated with Eosinophil Counts in Community-Dwelling Older Adults: A Cross-Sectional Study

Table S1. Complete 13-marker hematological screening correlations with serum α-Klotho.

Marker	n	Spearman ρ	p	BH q
Leukocytes	389	0.087	0.086	0.325
Neutrophils, absolute	388	0.070	0.166	0.360
Lymphocytes, absolute	388	0.056	0.272	0.393
Monocytes, absolute	388	0.116	0.022	0.146
Eosinophils, absolute	388	0.120	0.018	0.146
Basophils, absolute	388	0.013	0.792	0.858
Eosinophils, %	388	0.077	0.131	0.339
ESR	386	-0.057	0.261	0.393
NLR	388	0.017	0.732	0.858
PLR	388	-0.041	0.423	0.550
SII	388	0.007	0.883	0.883
SIRI	388	0.060	0.236	0.393
ELR	388	0.084	0.100	0.325

Note. The primary cohort was restricted to α-Klotho ≤6000 pg/mL. BH, Benjamini–Hochberg; ELR, eosinophil-to-lymphocyte ratio; ESR, erythrocyte sedimentation rate; NLR, neutrophil-to-lymphocyte ratio; PLR, platelet-to-lymphocyte ratio; SII, systemic immune-inflammation index; SIRI, systemic inflammation response index.

Table S2. Adjusted eosinophil-quartile and reverse-direction analyses.

Analysis	n	% difference	95% CI	p
Q2 vs Q1	376	10.5%	-4.4 to 27.8	0.177
Q3 vs Q1	376	14.2%	-0.4 to 31.0	0.058
Q4 vs Q1	376	18.7%	2.0 to 38.1	0.027
Reverse model: α-Klotho → eosinophils	376	19.1%	4.4 to 35.9	0.009

Note. Quartile estimates compare each category with Q1; p for linear trend across quartiles=0.019. The reverse model reports the percentage difference in eosinophil count per 1-SD higher $\log_{10}$(α-Klotho). All models used the fully adjusted covariate set.

Table S3. Stratified estimates and tests of effect modification.

Modifier	Stratum	n	% difference	95% CI	p interaction
Sex	Men	63	0.9%	-11.7 to 15.3	0.774
Sex	Women	313	9.0%	1.5 to 17.1	0.774
Age group	Age 60–69	242	8.3%	-0.0 to 17.4	0.908
Age group	Age ≥70	134	8.3%	-2.4 to 20.2	0.908
Respiratory disease	No respiratory disease	360	8.2%	1.7 to 15.3	0.632
Respiratory disease	Respiratory disease	16	14.7%	-19.3 to 62.9	0.632
eGFR category	eGFR ≥60	359	8.7%	2.2 to 15.6	0.868
eGFR category	eGFR <60	17	-5.8%	-52.3 to 85.8	0.868

Note. Estimates are the adjusted percentage difference in serum α-Klotho per 1-SD higher log-eosinophil count. Interaction p values come from product terms in the full primary cohort. Small strata yield wide confidence intervals.
